# Supplementary material for: Effect of Carbon Sources in Carotenoid Production from Haloarcula sp. M1, Halolamina sp. M3 and Halorubrum sp. M5, Halophilic Archaea Isolated from Sonora Saltern, Mexico
Source: Microorganisms. 2021 May 20;9(5):1096. doi: 10.3390/microorganisms9051096 (PMC8160830; doi:10.3390/microorganisms9051096)

S1. Detailed statistical analysis of carotenoid effect on pigment production at 120 h of culture.

Haloarcula sp. M1

BIOMASS

Analysis of Variance

Source	DF	Adj SS	Adj MS	F-Value	P-Value
Carbon source	4	0.008116	0.002029	13.32	0.001
Error	10	0.001523	0.000152		
Total	14	0.009639			

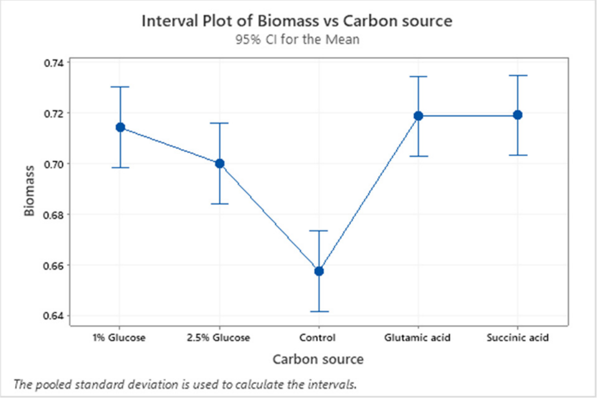

CAROTENOIDS

Analysis of Variance

Source	DF	Adj SS	Adj MS	F-Value	P-Value
Carbon source	4	218.615	54.6537	66.35	0.0000004
Error	10	8.237	0.8237		
Total	14	226.852			

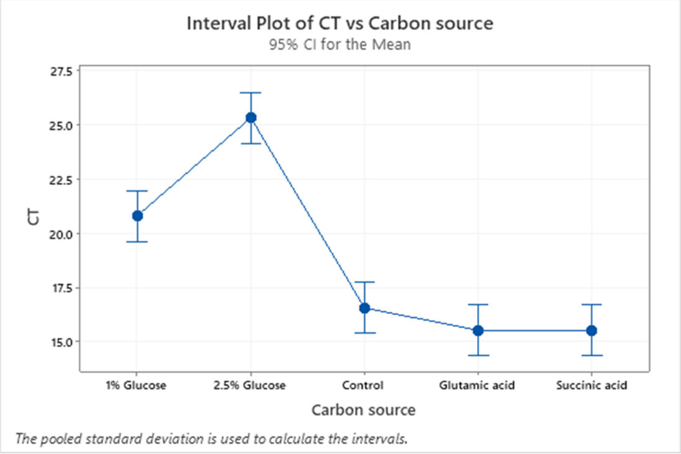

Halolamina sp. M3

BIOMASS

Analysis of Variance

Source	DF	Adj SS	Adj MS	F-Value	P-Value
Carbon source	4	0.002465	0.000616	1.34	0.322
Error	10	0.004614	0.000461		
Total	14	0.007078			

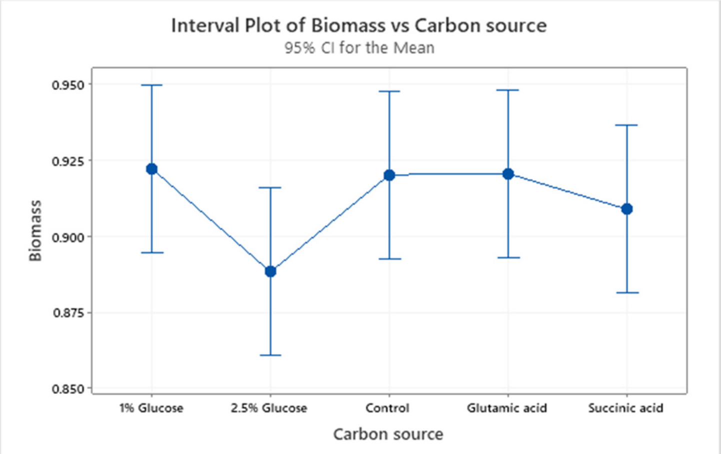

The pooled standard deviation is used to calculate the intervals.

CAROTENOIDS

Analysis of Variance

Source	DF	Adj SS	Adj MS	F-Value	P-Value
Carbon source	4	14.3318	3.58296	115.15	0.00000003
Error	10	0.3111	0.03111		
Total	14	14.6430			

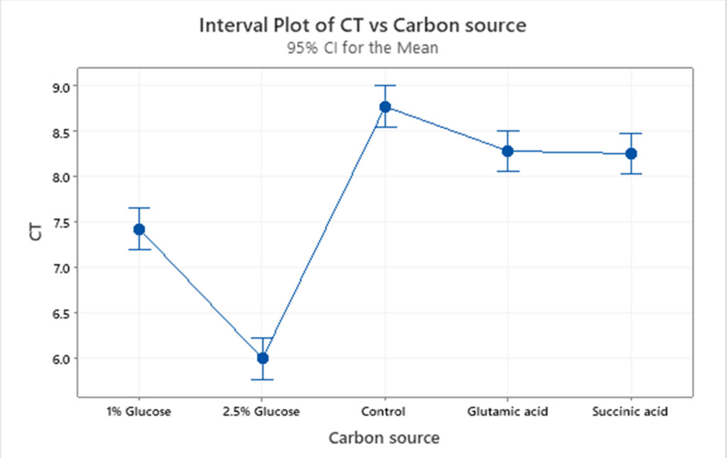

The pooled standard deviation is used to calculate the intervals.

Halorubrum sp. M5

BIOMASS

Analysis of Variance

Source	DF	Adj SS	Adj MS	F-Value	P-Value
Carbon source	4	0.183412	0.045853	691.68	0.000000000004
Error	10	0.000663	0.000066		
Total	14	0.184075			

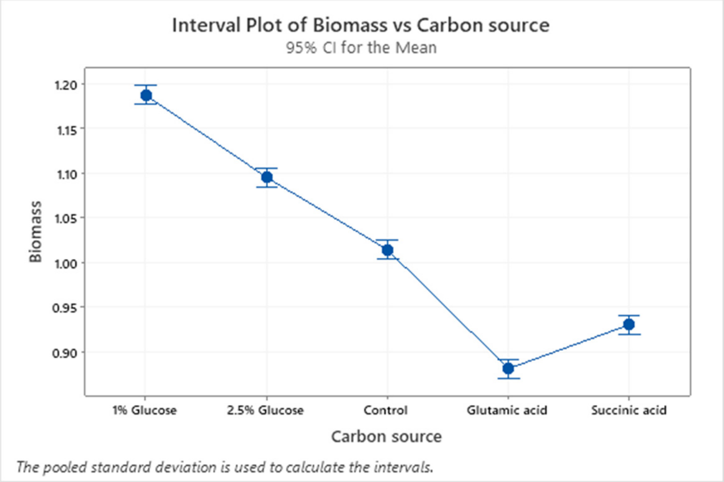

CAROTENOIDS

Analysis of Variance

Source	DF	Adj SS	Adj MS	F-Value	P-Value
Carbon source	4	142.051	35.5127	121.31	0.00000002
Error	10	2.928	0.2928		
Total	14	144.978			

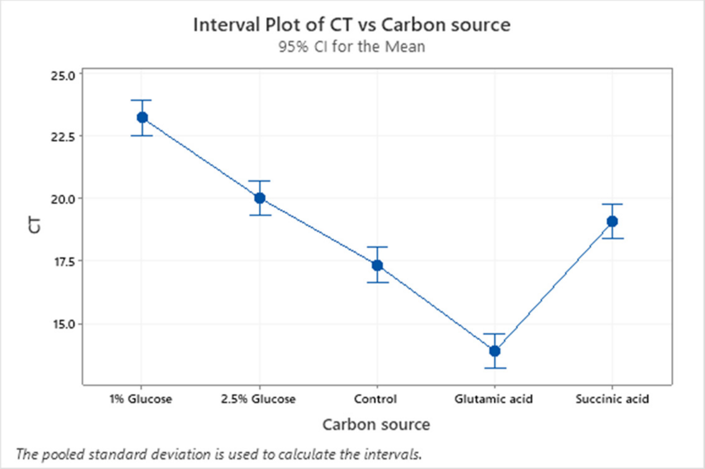

Supplement: Supplementary file 1 [file microorganisms-09-01096-s001.zip › microorganisms-1212290-supplementary.pdf]
